# Sympatric Occurrence of *Taenia solium, T. saginata,* and *T. asiatica,* Thailand

**DOI:** 10.3201/eid1309.061148

**Published:** 2007-09

**Authors:** Malinee T. Anantaphruti, Hiroshi Yamasaki, Minoru Nakao, Jitra Waikagul, Dorn Watthanakulpanich, Supaporn Nuamtanong, Wanna Maipanich, Somchit Pubampen, Surapol Sanguankiat, Chatree Muennoo, Kazuhiro Nakaya, Marcello O. Sato, Yasuhito Sako, Munehiro Okamoto, Akira Ito

**Affiliations:** * Mahidol University, Bangkok, Thailand; † Asahikawa Medical College, Asahikawa, Japan; ‡ Universidade Federal do Tocantins, Araguaína, Brazil; § Tottori University, Tottori, Japan; 1 These authors contributed equally to this article.

**Keywords:** Taenia asiatica, Taenia saginata, Taenia solium, sympatric distribution, dual infection, mitochondrial DNA analysis, Kanchanaburi, Thailand, dispatch

## Abstract

We confirmed sympatric occurrence of *Taenia solium, T. saginata,* and *T. asiatica* in western Thailand. DNA analysis of morphologically identified *T. saginata,* in a dual infection with *T. solium*, indicated it was *T. asiatica.* To our knowledge, this report is the first of *T. asiatica* and a dual *Taenia* infection from Thailand.

Taeniid tapeworm infections in the human intestine are caused by *Taenia solium, T. saginata,* and *T. asiatica* in Asia and the Pacific (*1*–*3*). Taeniasis caused by *T. solium* is a serious public health problem worldwide because eggs and proglottids expelled in the stool can infect humans through contamination of the environment and cause fatal neurocysticercosis. Neurocysticercosis cases caused by *T. solium* have increased in non–taeniasis-endemic areas (*3*–*5*).

A related taeniid tapeworm, Asian *Taenia* (= *T. asiatica*), has been described in Taiwan and the Republic of Korea (*1*–*3*,*6*–*8*). Although *T. asiatica* is phylogenetically closely related and is considered to be a sister species of *T. saginata* (*1*–*3*,*6*,*7*), the important intermediate host for *T. asiatica* is the domestic pig and the metacestodes mainly develop in the pigs’ liver (*6*). The morphologic characteristics of adult *T. asiatica* are very similar to those of *T. saginata*. Morphologic differentiation by either scolex or gravid proglottid of these 2 species is practically impossible (*1*,*3*). On the basis of molecular analysis of taeniid isolates from Asia and the Pacific, *T. asiatica* is distributed in Taiwan, the Republic of Korea, Malaysia, People’s Republic of China, Philippines, Indonesia, and Vietnam (*1*–*3*,*6*–*11*). However, there has been no evidence of the distribution of *T. asiatica* in Thailand (*8*,*12*).

## The Study

**Figure 1:**
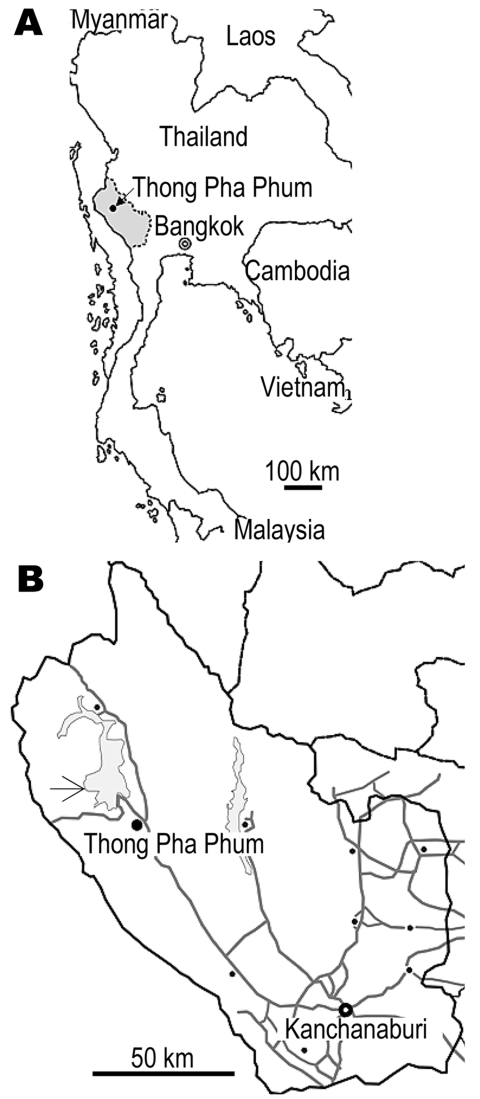
A) Map of Thailand showing Kanchanaburi Province (shaded area). B) The study area in Thong Pha Phum District (arrow).

The field investigation was conducted during 2002–2005 in communities in Thong Pha Phum District, 150 km northwest of Kanchanaburi Province, Thailand, close to the Myanmar border (Figure 1). The region is mountainous terrain that acts as a natural border between Thailand and Myanmar, and it mostly comprises natural parks and a water reservoir. The major studied population is Karen; Mon and Thai are minorities. In our study, molecular identification of these taeniid samples was conducted in Asahikawa, Japan in 2006. The study team, the Faculty of Tropical Medicine at Mahidol University, obtained ethical approval for a human stool survey for control of helminthic infections.

All taeniasis patients were informed of the study objectives and worm collection procedure through discussions, including about expulsion of proglottids. Tapeworms, either with scolices or without scolices, were expelled from 24 persons with taeniasis after the persons received 2 g of niclosamide or 40 mg/kg of praziquantel and a purgative. A total of 29 scolices were confirmed. Scolices with hooks expelled from 5 patients (nos. 2, 4, 7, 9, 11) were identified as *T. solium.* Scolices without hooks from 12 patients were identified as *T. saginata* because no molecular evidence on the distribution of *T. asiatica* in Thailand exists and all *Taenia* scolices without hooks were confirmed to be *T. saginata* in other areas in Thailand (*8*,*10*,*12*). Morphologic identification of the species was based on scolex only. Specimens without scolices were not identified morphologically. Most patients (12/17) harbored a single scolex. However, several patients harbored 2–6 scolices, including 1 dual infection with 2 *T. solium* and 1 *T. saginata* (patient 7) (Table). Patients were 7–60 years of age; 16 were male, and 8 were female.

**Table:** Characteristics of 24 taeniasis cases, Thailand, 2002–2005*

Patient				No. scolex/proglottids expelled	Morphologic identification of scolex	Preservative used	Molecular identification
No.	Sex	Age, y	Year				
1	M	60	2003	1 scolex without hook	*Taenia saginata*	NA	NT
2	F	34	2005	3 scolices with hooks	*T. solium*	NA	NT
3	M	55	2002	Segment without scolex†	–	Formalin	*T. solium*
4	M	29	2002	6 scolices with hooks	*T. solium*	Formalin	*T. solium*
5	F	38	2003	Segment without scolex†	–	Formalin	*T. solium*
6	F	46	2002	Segment without scolex†	–	Formalin	*T. solium*
7	F	28	2002	2 scolices with hooks, 1 scolex without hook	*T. solium,* *T. saginata*	AFA AFA	*T. solium* ***T. asiatica‡***
8	M	NK	2002	Segment without scolex†	–	Ethanol	*T. solium*
9	M	47	2004	1 scolex with hooks	*T. solium*	Ethanol	*T. solium*
10	M	7	2004	Segment without scolex†	–	Ethanol	*T. solium*
11	M	43	2004	3 scolices with hooks	*T. solium*	Ethanol	*T. solium*
12	M	10	2004	Segment without scolex†	–	Ethanol	*T. solium*
13	M	40	2003	Segment without scolex†	–	Ethanol	*T. saginata*
14	F	NK	2002	Segment without scolex†	–	Ethanol	*T. saginata*
15	M	40	2003	1 scolex without hook	*T. saginata*	Ethanol	*T. saginata*
16	M	55	2004	1 scolex without hook	*T. saginata*	Ethanol	*T. saginata*
17	F	30	2004	1 scolex without hook	*T. saginata*	Ethanol	*T. saginata*
18	M	13	2004	1 scolex without hook	*T. saginata*	Ethanol	*T. saginata*
19	M	45	2005	1 scolex without hook	*T. saginata*	Ethanol	*T. saginata*
20	F	42	2003	3 scolices without hook	*T. saginata*	Ethanol	***T. asiatica***‡§
21	F	28	2005	1 scolex without hook	*T. saginata*	Ethanol	***T. asiatica‡***
22	M	60	2005	1 scolex without hook	*T. saginata*	Ethanol	***T. asiatica‡***
23	M	32	2005	1 scolex without hook	*T. saginata*	Ethanol	***T. asiatica‡***
24	M	37	2005	1 scolex without hook	*T. saginata*	Ethanol	***T. asiatica‡***

*Year refers to year when specimen was collected; NA, not available; NT, not tested; Formalin, 10% formalin; AFA, alcohol-formalin-acetic acid; NK, not known; ethanol, 80% ethanol. †These segments without scolices were not examined for morphologic identification. ‡These cases (**boldface**) were identified as *T. saginata* morphologically but were confirmed to be *T. asiatica* by mitochondrial DNA analysis. §Three worms fixed separately.

Nineteen *Taenia* samples were fixed in 80% ethanol and kept at –20°C until use. Four samples were fixed in 10% formalin. All scolices were fixed with alcohol-formalin-acetic acid and stained with acetocarmine for morphologic comparative examination. One scolex with or without hooklets each from a patient 7 was further processed for molecular studies.

DNA samples were extracted from taeniid proglottids except for patient 7, for whom DNA was extracted from 2 scolices. DNeasy Blood and Tissue Kit (QIAGEN, Hilden, Germany) was used for the samples kept in ethanol. A DNA Isolator PS kit (Wako Pure Chemicals, Osaka, Japan) and DEXPAT (TaKaRa Shuzo, Shiga, Japan) were used for the formalin-fixed proglottids. DNA samples from 2 scolices expelled from patient 7 stained with acetocarmine were prepared by using DEXPAT and 0.05 N NaOH/1% sodium dodecyl sulfate containing proteinase K. Mitochondrial DNA diagnosis of ethanol-fixed samples was performed by multiplex PCR by using cytochrome *c* oxidase subunit 1 gene (*cox1*), except for the use of a forward primer (5′-TTATTTATTTACGTCAATCTTATTG-3′, positions 561–585) for *T. asiatica* (*10*). The formalin-fixed and acetocarmine-stained specimens were identified by base excision sequence scanning thymine-base (BESS T-base) analysis that used either *cox1* or cytochrome *b* gene (*cob*) (*13*). For BESS T-base analysis, the following primers were used: F3 (5′-TATTTGATCGTAAATTTAGTTCT-3′, corresponding to nucleotide (nt) positions 629–651) and R7 (5′-ATTAACACATAAACCTCGGGA-3′, nt positions 740–720) for *cox1* of *T. solium,* F1 (5′-GTCAAAAGATTCTTTTTTTACTTGGT-3′, nt positions 180–205) and R2 (5′-CCCTTCTTTCTATAACTTGAATAAT-3′, nt positions 305–281) for *cob* for *T. solium*. DNA sequencing of the products amplified by multiplex PCR was performed for confirmation. DNA samples for sequencing were prepared with a BigDye Terminator v3.1 Cycle Sequencing Kit (Applied Biosystems, Foster City, CA, USA). DNA sequencing was performed on an ABI PRISM 310 Genetic Analyzer (Applied Biosystems), and the nucleotide sequence data were analyzed by using DNASTAR version 3.75 (DNASTAR Inc., Madison, WI, USA).

**Figure 2:**
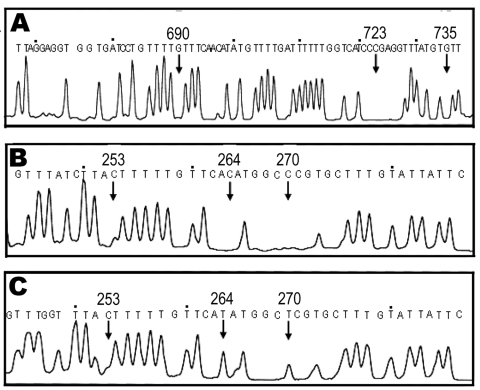
Molecular identification of formalin-fixed and acetocarmine-stained samples by base excision sequence scanning thymine-base (BESS) T-base analysis. BESS T-base profiles are shown in panels A, B, and C. A) Thymine-base profile using the 112-bp *cox1* products from patient samples 3–6. B) and C) BESS T-base analysis data that used 136-bp *cob* products from scolices with and without hooklets, respectively. Arrows indicate diagnostic positions. Nucleotide sequences indicated above the peaks are from GenBank databases (AB066485 for *cox1*, AB066570 and AB066580 for *cob*).

Multiplex PCR applied on 19 proglottids from 17 patients with *cox1* (*10*) showed that 5, 7, and 7 proglottids were *T. solium* (Asian genotype) (*10*,*13*), *T. saginata,* and *T. asiatica*, respectively (data not shown). These results were supported by DNA sequencing of the amplicons (data not shown). By contrast, small sizes of 112-bp *cox1* products were successfully amplified from samples taken from patients 3–6. These samples had been preserved in 10% formalin for years and BESS T-base analysis indicated that they were *T. solium* (Asian genotype) (Figure 2A) (*14*). BESS T-base analysis showed that scolices with and without hooklets from a dual infection (patient 7) were *T. solium* (Asian genotype) and *T. asiatica*, respectively (Figure 2B and C). To our knowledge, this is the first report demonstrating a dual infection with *T. solium* and *T. asiatica* in which 3 human taeniid cestodes are sympatrically distributed (*1*).

## Conclusions

We documented sympatric distribution of *T. solium*, *T. saginata,* and *T. asiatica* in western Thailand on the basis of mitochondrial DNA analysis. Our study indicated that 53.3% (8/15) of taeniid specimens expected to be *T. saginata* were *T. asiatica* and that both *T. asiatica* and *T. saginata* are codistributed in Kanchanaburi Province. Although *T. solium* taeniasis has seldom been reported in the literature in Thailand (*15*), our study has shown infection with *T. solium* (Asian genotype) in 11 (45.8%) of 24 *Taenia*-infected patients. The number of *T. solium* organisms expelled from taeniasis patients varied from 1 to 6, and >2 tapeworms were found in 36.4% (4/11) of *T. solium* taeniasis patients. In addition, we confirmed a dual infection with *T. solium* and *T. asiatica* (in patient 7). This experience indicates that molecular analysis is preferable and necessary for precise re-identification of so-called *T. saginata* in Asia and the Pacific (*1*).

Although *T. solium* cysticercosis in humans has not been reported in this study area, these populations appear to pose a risk for environmental contamination and person-to-person spread of *T. solium* leading to cysticercosis in humans and swine. Raw or inadequately cooked beef, pork, or pig viscera, and fresh blood are commonly consumed by local people in the study areas, and consequently they are at high risk of acquiring taeniasis. Therefore, to improve sanitation and quality of life, sustainable health education should be introduced and stressed to the population in the community.
